# Semi-Supervised Deep Image Stitching for Moving Elongated Objects

**DOI:** 10.3390/s26165092

**Published:** 2026-08-11

**Authors:** Xiao Lai, Ziqi Xie, Xianhui Liu

**Affiliations:** 1 School of Electronic and Electrical Engineering, Shanghai University of Engineering Science, Shanghai 201620, China; laixiao@sues.edu.cn; 2 College of Electronics and Information Engineering, Tongji University, Shanghai 200070, China; lxh@tongji.edu.cn

**Keywords:** deep image stitching, semi-supervised learning, image registration, image reconstruction

## Abstract

Image-stitching methods for moving elongated objects require high stitching quality, efficient inference, and robustness to interference from regions outside the target object. Existing methods still have difficulty satisfying these requirements simultaneously. This paper proposes a semi-supervised deep image-stitching method for moving elongated objects. The proposed framework consists of two stages: semi-supervised registration and unsupervised reconstruction. In the semi-supervised registration stage, a semi-supervised optical-flow estimation network is used to predict the bidirectional optical flow between the input images. An object-centric spatial transformation module is then introduced to remove regions outside the moving object and warp the inputs onto a unified plane. In the unsupervised reconstruction stage, a multi-scale fusion model is used to improve the quality of the reconstructed stitched image. Correspondingly, we design a reconstruction objective function based on multi-scale feature representations. To address the lack of available datasets for this task, we construct two datasets: MEOIS-D, a synthetic dataset for generalized evaluation, and Container-D, a real-world scene-specific dataset. Extensive comparative experiments and ablation studies demonstrate the effectiveness of the proposed method.

## 1. Introduction

Moving elongated objects, defined as entities with a long shape factor capable of locomotion, are ubiquitous in many industrial and transportation scenarios. Examples include trucks, railcars, shipping containers, and aircraft. Complete visual acquisition of such objects is important for downstream analysis and inspection. For instance, in traffic monitoring, complete images of railcars and trucks are useful for fault diagnosis tasks [1,2]. In port production, complete shipping container images are vital for damage assessment and defect detection [3,4]. In aviation inspection, full aircraft images support comprehensive condition analysis and fault diagnosis [5,6].

However, obtaining a complete image of a moving elongated object remains challenging. These objects often have specific features such as unstable movement speed and long length, which render general photographic equipment incapable of capturing them in their entirety in a single shot. Therefore, it is typically necessary to use image-stitching algorithms to fuse multiple local images of a moving elongated object.

**Figure 1:**
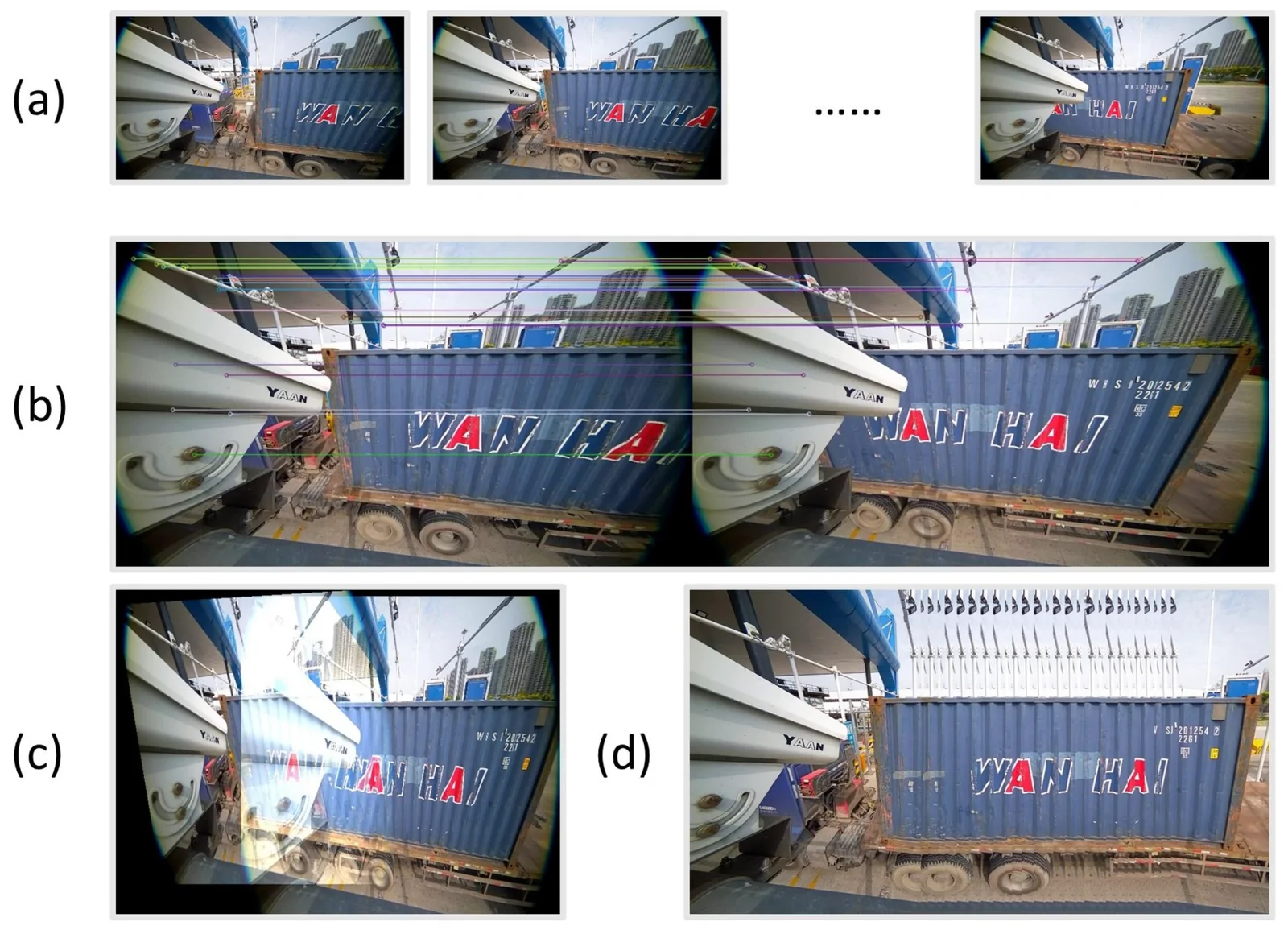
An example of stitching a container image using current image-stitching methods. (**a**) Input image frames. (**b**) Matching images by SURF [10] and RANSAC [11], we present the top-25 best matching pairs, and it is evident that the presence of both background and foreground regions greatly hinders the use of classical matching algorithms. (**c**) Stitched image using a panoramic image-stitching method [9]. A foreground camera artifact appears in the stitched image. (**d**) Stitched image using an image-stitching method for moving elongated objects [12]. The presence of perspective distortion results in evident deformation in the stitched image. Also, the characters in the container’s upper-right corner are noticeably incomplete due to inaccuracies in the matching algorithm.

Based on the target application, existing image-stitching methods can be broadly divided into panoramic image-stitching methods and moving elongated object image-stitching methods. Panoramic image-stitching methods [7,8,9] typically estimate a homography between images, warp the images according to the estimated transformation, and fuse the overlapping regions. These methods usually assume that the full image is the stitching target. As shown in Figure 1b, registration in such methods can be strongly affected by foreground and background interference. When directly applied to moving elongated objects, foreground occlusions, such as the camera shown in Figure 1, may be incorrectly preserved in the stitched result. Therefore, without object-level segmentation or filtering, existing panoramic image-stitching methods cannot reliably process images of moving objects.

Image-stitching methods for moving elongated objects [12,13,14,15] usually capture multiple frames of a moving object, estimate the object displacement between frames, crop corresponding image regions, and then combine the cropped regions into a complete object image. These methods are often restricted to specific imaging angles. When the input images contain obvious perspective distortion, stitching errors increase substantially. Moreover, because such methods often require dozens of frames, their computational cost is relatively high.

Figure 1 shows an example of stitching a container image to demonstrate the limitations of current methods. By analyzing the limitations of existing methods, we believe there are three issues that need to be addressed in image-stitching methods for moving elongated objects. First, the performance of image-matching algorithms decreases substantially when input images contain cluttered foreground or background regions. Consequently, existing methods may produce visible deformation when the target object exhibits perspective distortion. Finally, traditional moving object image-stitching methods based on many frames are often time-consuming.

To address these issues, we propose a semi-supervised deep image-stitching method for moving elongated objects. The framework consists of semi-supervised registration and unsupervised reconstruction. Rather than treating each component as an isolated novelty, the main contribution of this work is an object-centric image-stitching framework tailored to moving elongated objects. The framework integrates semi-supervised optical-flow registration, object-level spatial transformation, and multi-scale reconstruction to address foreground/background interference, perspective distortion, and efficient pairwise stitching.

The main contributions of this article are summarized as follows:

- We propose a semi-supervised optical-flow network to improve matching accuracy in the image registration stage. Compared with homography-based panoramic image stitching, dense optical flow provides object-level motion estimation that is more suitable for objects affected by perspective distortion and foreground/background interference.
- We design an object-centric image spatial transformation module. This module removes regions outside the moving object and maps the input images onto a unified plane using the predicted optical flow. Specifically, a coarse-to-fine warping strategy is introduced, which maps images onto a unified plane, while refined warping suppresses residual misalignment and unreliable warped regions.
- We formulate the fusion stage as object-centric image reconstruction. A U-Net-based reconstruction network with multi-scale feature fusion is used to enhance local details, seam continuity, and overall visual quality in the stitched object image.
- To address the lack of available datasets for this task, we construct two datasets. MEOIS-D is a synthetic dataset generated with Blender [16], covering six object categories, multiple lighting environments, and different overlap levels. Container-D is a real-world dataset collected from container-truck images in port production scenarios. Experiments on both datasets demonstrate the effectiveness of the proposed method.

## 2. Related Works

In this section, we review the key image-stitching techniques: panoramic image-stitching methods and image-stitching methods for moving elongated objects.

### 2.1. Panoramic Image Stitching

Traditional panoramic image-stitching methods are mainly feature-based. A classical solution [11] uses a global homography to register and fuse input images. However, when images contain multiple depth layers, such methods may produce visible ghosting artifacts. Various techniques have therefore been proposed to reduce these artifacts. DHW [17] assumes two planes in the images and applies different homographies to each plane. APAP [18] divides an image into a dense grid and aligns different regions using spatially adaptive warping. Seam-driven image stitching [19] selects an optimal homography by minimizing seam-cutting loss. Lin et al. [20] use iterative warping and seam estimation to preserve curves and lines during stitching. Nevertheless, feature-based methods rely heavily on feature detection and may perform poorly in low-texture, repetitive-pattern, or low-visibility scenes.

With the development of deep learning, learning-based panoramic image stitching has attracted increasing attention. Early deep image-stitching methods either had limited generalization to specific scenes and fixed viewpoints [21,22] or used deep learning only for feature extraction without forming a complete learning-based image-stitching framework [23,24]. VFISNet [25] stitches images captured from arbitrary viewpoints using a deep model, but it can process only images of fixed size. EPISNet [8] introduces a large-baseline deep homography module to estimate projective transformations at different scales and an edge-preserving deformation module to reduce artifacts. However, these methods are supervised, which limits their transferability to real-world environments. The authors of [9] address this limitation by proposing UDIS, an unsupervised deep image-stitching framework that can be trained using unlabeled real-world data. UnsupDIS-YOLO [26], a variant of UDIS based on YOLOv5 [27], improves computational efficiency. UDIS++ [7] further optimizes the performance of UDIS. SRStitcher [28] introduces a generative-model-based image-stitching architecture, yet the inherent uncertainty of generative models remains a major concern. However, existing panoramic image-stitching methods are not designed for moving elongated objects and do not explicitly address foreground and background interference around the target object.

### 2.2. Moving Elongated Object Image Stitching

Compared with panoramic image-stitching methods, relatively little research has focused on image-stitching methods for moving elongated objects. Most existing methods rely on traditional approaches and are designed for specific application scenarios. For example, Nie et al. [29] proposed a 3D reconstruction method for train accident scenes based on SIFT and RANSAC [11], but the method requires camera calibration, limiting its applicability. Diverting Pan [13] designed a train image-stitching solution based on traditional sparse optical-flow estimation. Bahrami et al. [4] introduced an optical-flow-based image-stitching method for port container images, but the detailed design of the optical-flow estimation method was not specified.

Several works [12,14,15] focus on port container stitching and rely on traditional feature detection, such as SURF [10] and SIFT. Their main differences lie in their matching strategies. These methods typically use multiple frames to calculate inter-frame offsets. As discussed above, existing image-stitching methods for moving elongated objects often require strict imaging angles. When perspective distortion occurs, the stitched image can exhibit obvious deformation. In addition, traditional registration methods may fail to accurately match moving elongated objects and can even lead to computational failure, resulting in lower stitching quality. These limitations are further demonstrated in the Experiments section. The key challenge addressed in this paper is object-centric geometric alignment and reconstruction under foreground/background interference and perspective distortion.

## 3. Method

**Figure 2:**
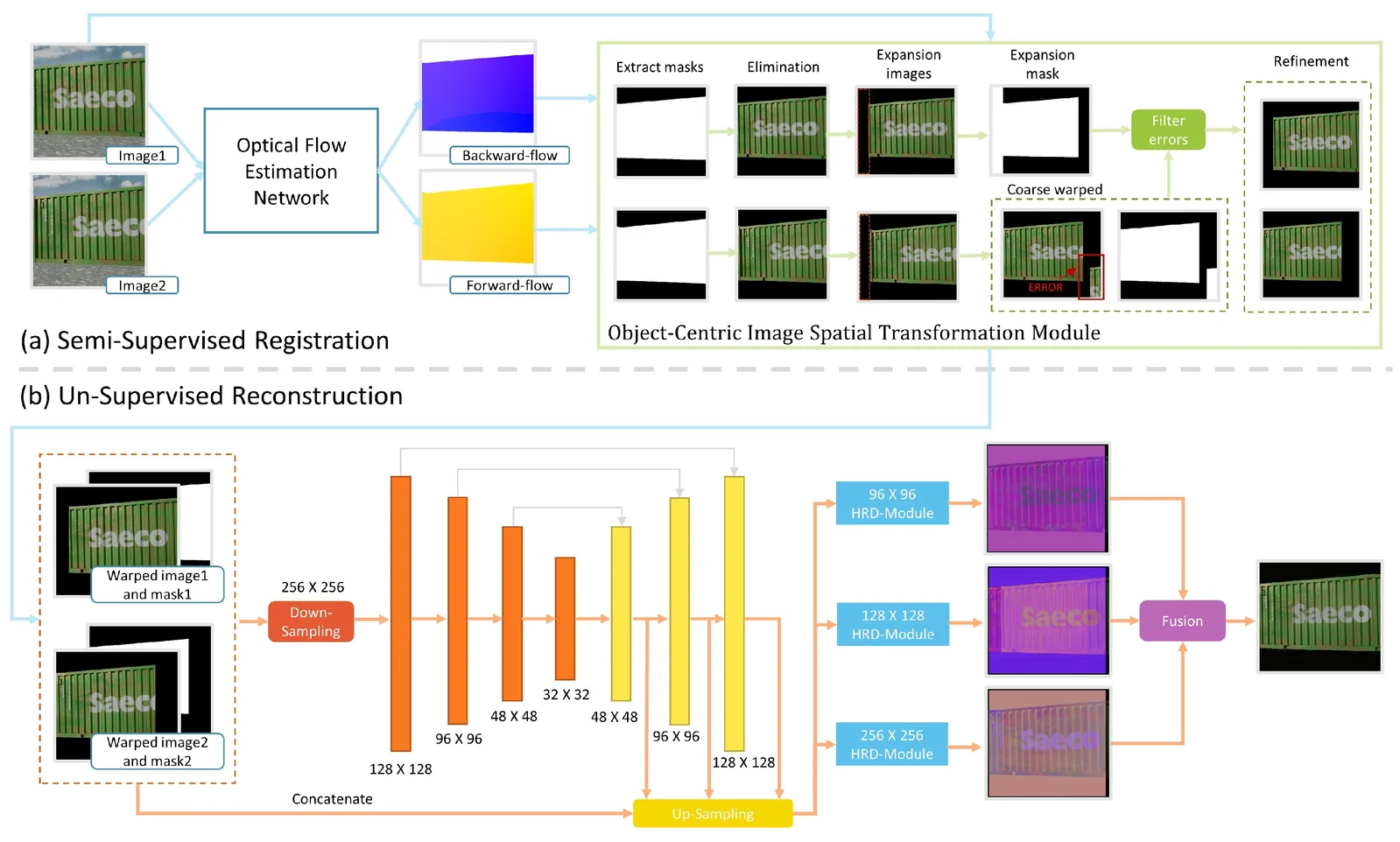
Overview of the proposed semi-supervised deep image-stitching framework for moving elongated objects. The input image pair is processed in two stages. (**a**) Semi-supervised registration estimates the bidirectional optical flow between the input images. An object-centric image spatial transformation module removes regions outside the moving object and warps the inputs onto a unified plane. (**b**) Unsupervised reconstruction takes the warped image pair as input and generates the stitched image. By formulating image stitching as a reconstruction task, the network uses a U-Net architecture and multi-scale fusion to improve visual quality. Features at different scales emphasize different details, and their fusion provides richer information than single-scale reconstruction.

Figure 2 shows the overall framework of the proposed method. The framework comprises two stages: an optical-flow estimation network for image alignment and an image reconstruction network for image fusion. Between these two stages, an object-centric image spatial transformation module removes regions outside the moving object and warps the inputs onto a unified plane.

The two-stage formulation is adopted because image stitching for moving elongated objects involves two different but coupled subproblems: object-centric geometric alignment and appearance-level image reconstruction. This decomposition is consistent with the general design of image-stitching pipelines, where registration/warping and fusion/blending are usually treated as separate functional stages. Similar modular designs are also common in learning-based image-stitching methods. For example, EPISNet estimates a global transformation and then applies deformation refinement [8], while UDIS [9] and UDIS++ [7] formulate unsupervised stitching by first aligning the image content and then reconstructing the stitched features into images.

To reduce registration errors, the proposed object-centric image spatial transformation module is placed between the two stages to reduce error propagation. Specifically, the module converts bidirectional optical flow into object-centric masks, removes non-object regions that are likely to contain irrelevant foreground or background interference, and uses coarse-to-fine warping to reduce residual misalignment before reconstruction. As a result, the reconstruction network mainly receives object-centered aligned content rather than a full image pair containing large unreliable regions. In our task, this separation is particularly useful because the registration stage suppresses foreground/background interference and maps the moving object to a unified plane, whereas the reconstruction stage focuses on seam continuity, texture consistency, and visual quality.

### 3.1. Semi-Supervised Registration

Suppose the input images are $I_{A}$ and $I_{B}$$\in R^{H\times W\times 3}$, where *H* and *W* are the image height and width, respectively. We need to estimate the bidirectional optical flow $f_{B}^{A},f_{A}^{B}=\left(u,v\right)\in R^{H\times W\times 2}$ between the two images. To improve computational efficiency, we introduce a network structure [30] that can estimate the bidirectional optical flow in a single inference.

Creating labeled dense matching datasets is costly, particularly under real-world conditions. Therefore, we use a training method that does not require labeled data. There are two options: an unsupervised method or a semi-supervised method.

While many recent works have advanced unsupervised optical-flow estimation [31,32], current methods remain highly parameter-sensitive. And using only the unsupervised loss function to fine-tune the network usually results in a poorer local minimum [33]. Fully unsupervised methods still underperform compared with supervised methods on optical-flow benchmarks [34], demonstrating the limitations of unsupervised methods.

In contrast, some recent semi-supervised optical-flow methods have demonstrated exceptional performance [33,34]. These advancements motivate the use of a semi-supervised approach to fine-tune our optical-flow estimation network.

Inspired by previous work [35], we alternately train on labeled and unlabeled data. The objective function of the optical-flow estimation network consists of two parts: the supervised loss $L_{sup}$ and the unsupervised loss $L_{unsup}$.

Suppose there are *N* estimated optical flows, including the intermediate and final ones. And the *i*-th estimated flow is $f_{i}$, and the corresponding ground-truth flow is ${\hat{f}}_{i}$.

#### 3.1.1. Supervised Loss

For labeled data, we calculate the supervised loss $L_{sup}$, as follows:

(1) $$L_{sup}^{i}={∥f_{i}-{\hat{f}}_{i}∥}_{1}$$

#### 3.1.2. Unsupervised Loss

For unlabeled data, we calculate the unsupervised loss $L_{unsup}$, as follows:

(2) $$L_{unsup}^{i}=\rho \left(warp\left(I_{B}^{i},f_{i}\right)-I_{A}^{i}\right)$$

where ρ(·) is the Charbonnier loss [31] and warp(·) is a warping function that uses bilinear sampling to warp $I_{B}$ based on the estimated flow $f_{i}$.

In conclusion, the objective function of the optical-flow estimation network is as follows:

(3) $$L_{Regis}=\sum_{i=1}^{N}\gamma^{N-i}\left(\lambda_{sup}L_{sup}^{i}+\lambda_{unsup}L_{unsup}^{i}\right)$$

where γ is an exponential weighting factor, which, following previous work [30], is set to 0.9, and $\lambda_{sup}$ and $\lambda_{unsup}$ are the weights corresponding to the two loss functions, set to 1 and 0.1, respectively.

### 3.2. Object-Centric Image Spatial Transformation Module

The object-centric image spatial transformation module is designed to remove regions outside the moving object and warp images onto a unified plane. This module also reduces the influence of inaccurate optical flow outside the target object. In image-stitching methods for moving elongated objects, non-object regions are usually irrelevant to the final stitched target and may contain foreground/background interference. Directly feeding these regions into the reconstruction network would increase the risk of artifacts. Therefore, a coarse-to-fine warping approach is used to exclude unreliable warped regions from the reconstruction input.

#### 3.2.1. Coarse Warping

The object masks $M_{1}^{o}$ and $M_{2}^{o}$ are easily obtained from $f_{B}^{A}$ and $f_{A}^{B}$. Then, the object-centric images $I_{A}^{\prime }$ and $I_{B}^{\prime }$ can be acquired by $I_{A}⊙M_{1}^{o}$ and $I_{B}⊙M_{2}^{o}$. To maintain the integrity of the warped images, we need to leave redundant space, represented by $space\in R^{H\times D\times 3}$, where *D* is set to $max(\left|\left\lceil v\right\rceil \right|)$. Thus, the expanded images are $I_{A}^{\prime ex},I_{B}^{\prime ex}\in R^{H\times (W+D)\times 3}$. After that, the coarse warped image $I_{B}^{coarse}=warp\left(I_{B}^{\prime ex},f_{B}^{A}\right)$ is obtained.

#### 3.2.2. Refined Warping

It is important to note that optical-flow estimation is not completely accurate and may produce mapping errors, particularly outside the object mask range. To address this issue, we create a filter to remove incorrect warp areas. We generate an expansion mask $M_{1}^{ex}\in R^{H\times (W+D)\times 3}$ based on $M_{1}^{o}$ in a similar way to that used for generating expansion images. The extension mask sets the area with width *D* on the left to be reserved and the area with width *D* on the right to be filtered. Then, the refined warped image $I_{B}$ is obtained by $I_{B}^{coarse}⊙M_{1}^{ex}$.

Finally, we get the object-centric and unified plane images $I_{A}$ (that is, $I_{A}^{\prime ex}$) and $I_{B}$. We provide a detailed visualization of this process in Figure 2.

### 3.3. Unsupervised Reconstruction

Using the image reconstruction network to generate stitched images essentially transforms the image fusion problem in traditional image stitching into a reconstruction problem.

The inputs of the unsupervised reconstruction network are the images $I_{A},I_{B}$ and their corresponding content masks $M_{A}^{c},M_{B}^{c}$. The input masks do not participate in the forward propagation of the network. They are utilized to calculate the seam masks $M_{A}^{s},M_{B}^{s}$ based on the method in [9]. The content masks and seam masks are further used in the objective function.

The input images are first downsampled to 256×256 by a focus layer. Then, a UNet structure network is utilized to reconstruct the low-resolution stitched image. The encoder is composed of four combinations of Convolution(Conv)+C3 layers, with the convolutional kernels set to {64, 64, 128, 128, 256, 256, 512, 512}. The decoder is made up of three combinations of Conv+Upsample+C3 layers, with the convolutional kernels set to {256, 256, 128, 128, 64, 64} (the upsample layer without a convolutional kernel). It should be noted that the focus and C3 layers are from YOLOv5 [27], and the upsample layer performs upsampling through bilinear interpolation.

From the decoder, we obtain the multi-scale features $F_{up}^{\frac{1}{2}},F_{up}^{\frac{1}{4}},$ and $F_{up}^{\frac{1}{8}}$, which are upsampled to a size of 256×256 through convolution and interpolation to obtain the low-resolution stitched images $S_{LR}^{1},S_{LR}^{2},$ and $S_{LR}^{3}$, respectively. The low-resolution stitched images are upsampled and concatenated with the original input images and then fed into three HR deformation modules, producing three multi-scale high-resolution stitched images $S_{HR}^{1},S_{HR}^{2},$ and $S_{HR}^{3}$, respectively. Eventually, the fusion module fuses the $S_{HR}^{1},S_{HR}^{2},$ and $S_{HR}^{3}$ images into the final output image $S_{HR}$. Both the fusion module and the HR deformation module are composed of one combination of Conv+C3+Conv layers, with the convolutional kernels set to {64, 64, 3}.

The objective function of the unsupervised reconstruction network consists of three parts, as detailed below.

#### 3.3.1. Low-Pixel Loss

The low-pixel loss is composed of the low-pixel content loss $L_{content}^{low}$ and the low-pixel seam loss $L_{seam}^{low}$. $L_{content}^{low}$ is used to constrain the features and content fidelity of the stitched image, whereas $L_{seam}^{low}$ is used to constrain the natural continuity of the overlapping area edges.

To calculate $L_{content}^{low}$, it is necessary to compute the perceptual loss $L_{P}$ of each low-resolution stitched image. Taking $S_{LR}^{1}$ as an example, the perceptual loss of $S_{LR}^{1}$ is $L_{P}^{1}$, as follows:

(4) $$L_{P}^{1}=\sum_{i\in \{A,B\}}MSE\left(\phi_{j}\left(S_{LR}^{1}⊙M_{i}^{c}\right),\phi_{j}\left(I_{i}^{256\times 256}\right)\right)$$

where MSE is the mean square error and $\phi_{j}\left(\cdot \right)$ denotes extracting the feature representations of $S_{LR}^{1}⊙M_{A}^{c}$ and $I_{A}$ by VGG-19 [36] at the *j*-th layer (set to conv5_3).

The calculation of the other low-resolution stitched image perceptual losses is similar. $L_{content}^{low}$ is the sum of three perceptual losses, that is, $L_{content}^{low}=\sum L_{P}^{i},i\in \left\{1,2,3\right\}$.

To calculate $L_{seam}^{low}$, we must compute the single seam loss of each low-resolution stitched image. Taking $S_{LR}^{1}$ as an example, the single seam loss of $S_{LR}^{1}$ is $L_{S}^{1}$, as follows:

(5) $$L_{S}^{1}=\sum_{i\in \{A,B\}}{∥\left(S_{LR}^{1}⊙M_{i}^{s},I_{i}^{256\times 256}⊙M_{i}^{s}\right)∥}_{1}$$

Thus, $L_{seam}^{low}=\sum L_{S}^{i},i\in \left\{1,2,3\right\}$.

Above all, the low-pixel loss is defined as

(6) $$L_{low}=\lambda_{content}L_{content}^{low}+\lambda_{seam}L_{seam}^{low}$$

where $\lambda_{content}$ and $\lambda_{seam}$ are the weights of $L_{content}^{low}$ and $L_{seam}^{low}$, respectively. In our experiments, $\lambda_{content}$ and $\lambda_{seam}$ are set to 1 and 10.

#### 3.3.2. High-Pixel Loss

We calculate the high pixel-level loss $L_{high}$ solely from the final output $S_{HR}$, which is defined similarly to the low-pixel loss. The key distinction lies in using the conv3_3 layer of VGG-19 to compute the perceptual loss. Consequently, the $L_{high}$ can be expressed as

(7) $$L_{high}=\lambda_{content}L_{content}^{high}+\lambda_{seam}L_{seam}^{high}$$

#### 3.3.3. Content Consistency Loss

To eliminate parallax artifacts, we incorporate the content consistency loss $L_{consistency}$, which is defined as

(8) $$L_{consistency}=\sum_{i\in \{1,2,3\}}{∥S_{HR}^{i}-S_{LR}^{i}∥}_{1}$$

Conclusively, the objective function $L_{Recon}$ for the image reconstruction network is derived as follows:

(9) $$L_{Recon}=\lambda_{low}L_{low}+\lambda_{high}L_{high}+\lambda_{consistency}L_{consistency}$$

where $\lambda_{low}$, $\lambda_{high}$, and $\lambda_{consistency}$ are the weights of the three losses, which are set to 100, 1, and 1 in the experiments, respectively.

**Figure 3:**
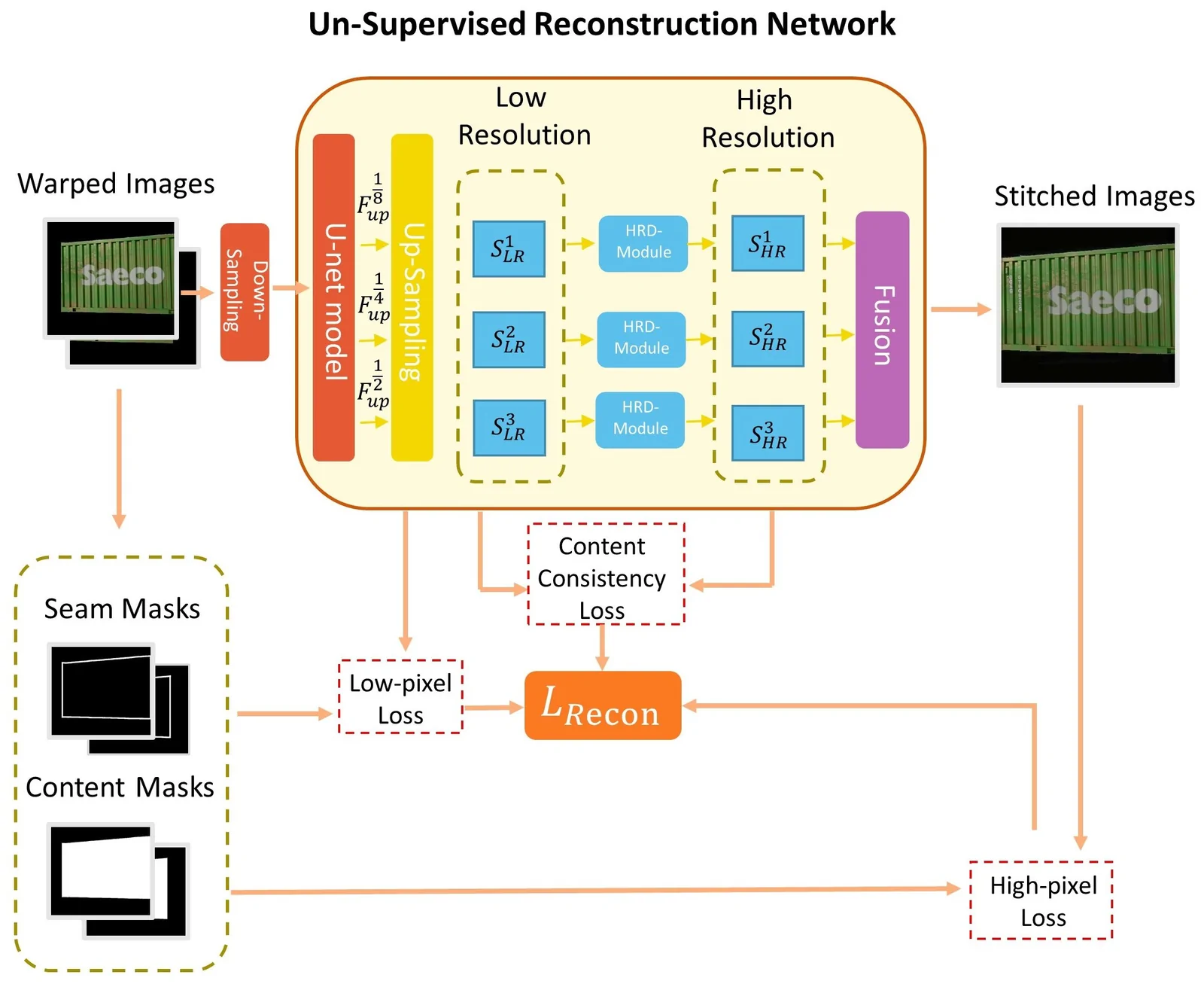
Visualization of the calculation of the objective function in our unsupervised reconstruction network.

For clarity, we present a visualization of the calculation of the objective function in Figure 3.

## 4. Experiments

In this section, we conduct extensive experiments to validate the effectiveness of the proposed method.

### 4.1. Datasets

To evaluate the generalization ability of the proposed method, we construct the moving elongated object image-stitching dataset (MEOIS-D) using the 3D simulation software Blender [16]. To ensure diversity, the dataset contains different lighting environments and multiple object categories. We use freely shared model packages from BlenderKit [37] to generate different object types. Synthetic data generation with Blender has become a common strategy in deep learning research when real data collection is difficult or costly.

**Figure 4:**
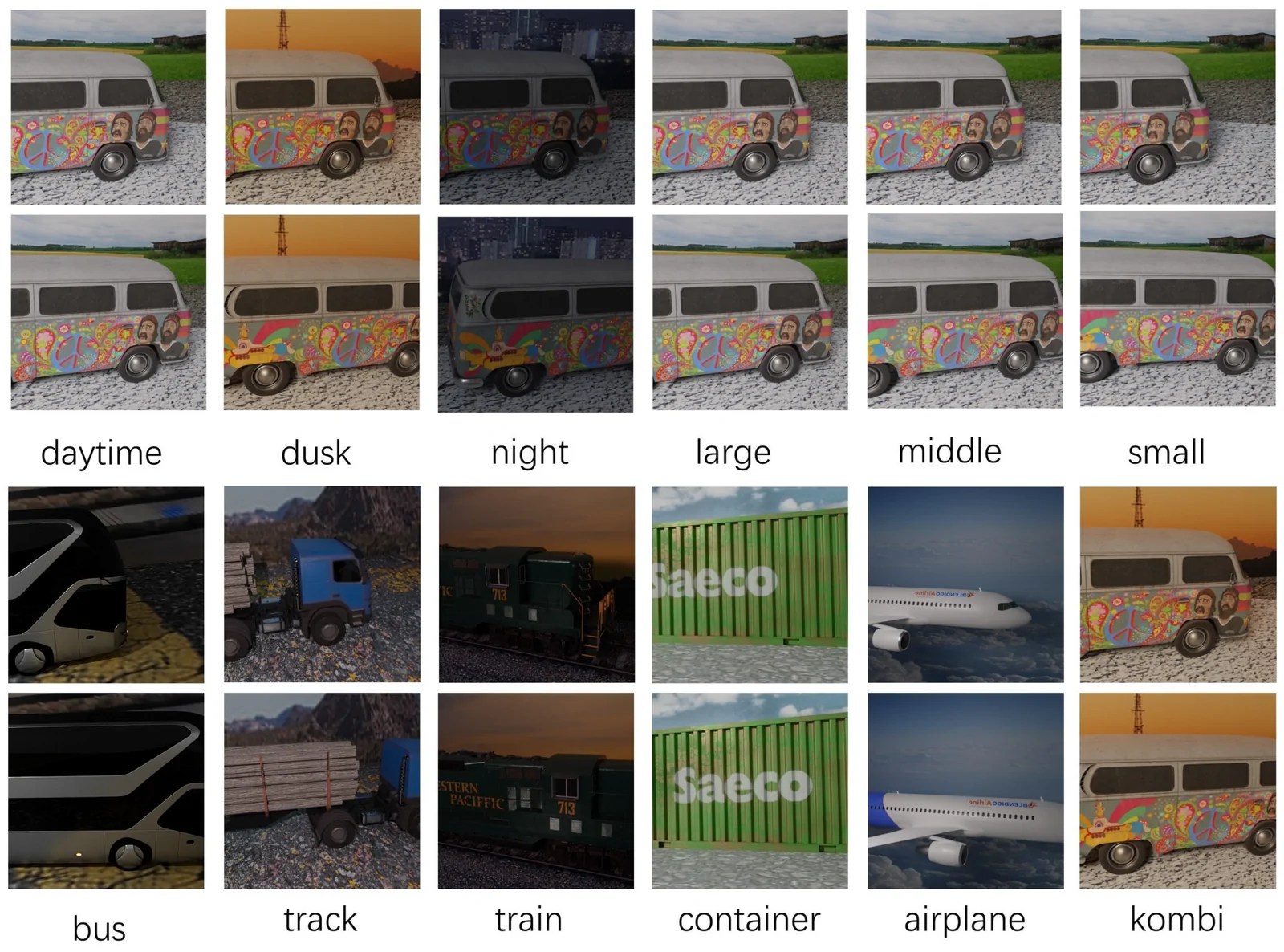
Images from MEOIS-D. The images in the first three columns represent the three different lighting environments: daytime, dusk, and night. The images in the last three columns represent the three overlap levels. Readers can see the differences in the images by focusing on the lemon icon on the kombi. We also present the different types of moving elongated objects, covering sea (a shipping container), land (various types of transport vehicles, including a bus, a truck, a train, and a kombi), and air (an airplane).

We first fix the camera position and then randomly move and rotate the object to capture pairs of consecutive images with different overlap levels. For an object with length length, three overlap levels are defined, as shown in Table 1. To improve realism, we collect a large number of real-world images as background textures. Color jittering is then applied to increase variations in illumination and object appearance. MEOIS-D contains 5097 image pairs of size 512×512. The training-to-testing ratio is approximately 10:1. The dataset is balanced across three overlap levels and three lighting environments, with a ratio of approximately 1:1:1 in each case. It includes multiple moving elongated objects, including buses, trucks, trains, containers, airplanes, and kombis. Although the kombi is less elongated than objects in some other categories, it has complex body patterns that make image-stitching coherence challenging. It is therefore included as a difficult case for evaluating the applicability of the proposed method. Example images from MEOIS-D are shown in Figure 4.

**Figure 5:**
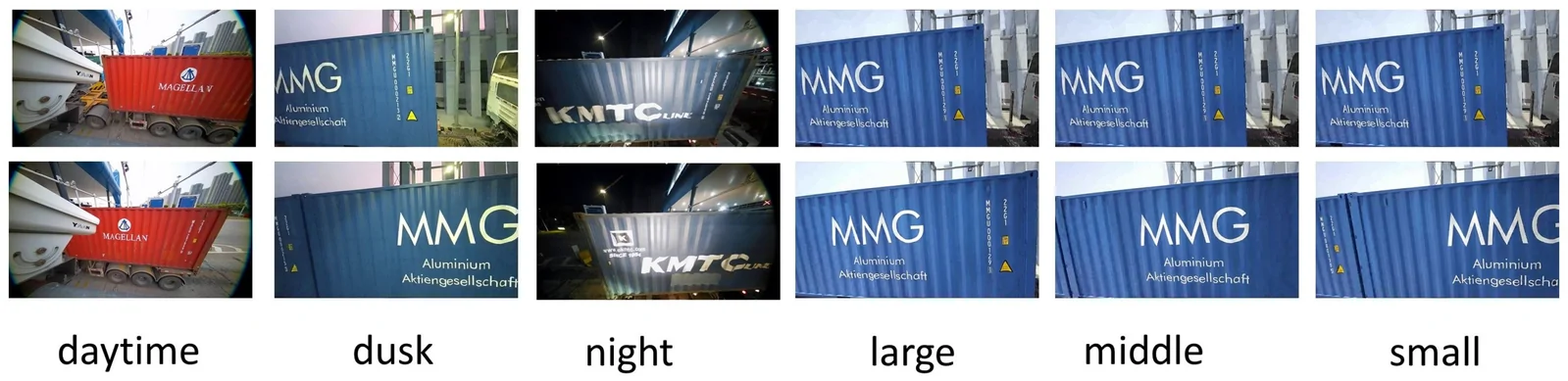
Images from Container-D. The images in the first three columns represent the three different lighting conditions: daytime, dusk, and night. The images in the last three columns represent the three overlap levels. Readers can see the differences in the images by focusing on the text *MMG*.

To evaluate real-world performance, we construct Container-D using container-truck frame data collected by surveillance cameras at the gate of Shanghai Port during different time periods. Container-D contains 5000 image pairs of size 960×540, with a training-to-testing ratio of approximately 10:1. The dataset is divided into three overlap levels: large, medium, and small, corresponding to frame intervals of 3, 5, and 8, respectively. The three overlap levels are approximately balanced. The dataset also covers three lighting environments: daytime, dusk, and night. Example images from of Container-D are shown in Figure 5.

### 4.2. Implementation Details

The proposed method is trained in two stages. In the first stage, the optical-flow estimation network is initially pre-trained on FlyingChairs [38] for 100K iterations with a batch size of 16 and a learning rate of $4\times 10^{-4}$. It is then pre-trained on FlyingThings3D [38] for 200K iterations with a batch size of 8 and a learning rate of $2\times 10^{-4}$.

The optical-flow estimation network is then fine-tuned using semi-supervised learning. Specifically, supervised training is performed on Sintel [39], while unsupervised training is performed separately on MEOIS-D and Container-D. The fine-tuning process uses 100K iterations, a batch size of 1, and a learning rate of $1\times 10^{-5}$. In the second stage, the unsupervised reconstruction network is trained separately on MEOIS-D and Container-D for 150K iterations, with a batch size of 4 and a learning rate of $3\times 10^{-4}$. Adam is used as the optimizer. The framework is implemented in PyTorch2.0, and training and testing are performed on four NVIDIA GTX 1080Ti GPUs.

### 4.3. Evaluation of Registration

We compare our method with several registration methods. These methods can be broadly divided into two categories:

1. Traditional Registration Methods: SURF+RANSAC(S+R) [11], the most commonly used method, which is widely used in related works [12,14], and SURF+MAGSAC (S+M) [40]—MAGSAC is a more accurate solution than RANSAC proposed in recent years.
2. Learning-based Registration Methods: UDIS [9], a pioneering deep image-stitching method; UDIS-Variant [26], which improves both the registration model and loss function in UDIS (although UDIS-Variant has not been published in any paper, its results show that its registration performance is significantly better than that of UDIS); CA-UDHN [41], a classic unsupervised homography model; SMURF [42], a classic unsupervised optical-flow model; and FlowSupervisor (FS) [33], a groundbreaking semi-supervised optical-flow model.

Following previous work [9], we utilize $P_{overlap}$ and $S_{overlap}$ as our metrics. Specifically, assuming that the image is $I_{A}$, the warped image is $I_{B}$, and the overlap mask is $M_{overlap}=M_{A}^{c}⊙M_{B}^{c}$, the calculations of $P_{overlap}$ and $S_{overlap}$ are

(10) $$\begin{array}{c} P_{overlap}=PSNR\left(M_{overlap}⊙I_{A},M_{overlap}⊙I_{B}\right),\\ S_{overlap}=SSIM\left(M_{overlap}⊙I_{A},M_{overlap}⊙I_{B}\right) \end{array}$$

where PSNR(·),SSIM(·) denote the operations of computing PSNR and SSIM [43] between two inputs.

Comparisons between our method (denoted as Ours) and other algorithms are shown in Table 2, Table 3, Table 4 and Table 5. Our method outperformed all other approaches regarding the different overlap levels and lighting environments. It also achieved the best or second-best performance on most object types, with overall superior performance. Notably, traditional methods may encounter computational errors, such as an insufficient number of detected matching pairs and an invalid homography matrix, resulting in the need to skip some samples during testing and highlighting the limitations of traditional methods.

Furthermore, a counterintuitive result is that the metric values under night lighting conditions are higher than those under daytime and dusk conditions. Our analysis suggests that the purity, brightness, and contrast of night images are lower than those of the other two environments. These factors are beneficial for improving the PSNR and SSIM scores, but they do not necessarily mean that matching night images is easier.

### 4.4. Evaluation of Reconstruction

In the previous subsection, only UDIS and UDIS-Variant proposed reconstruction schemes among the methods compared, while the other methods had only the registration stage without reconstruction. Therefore, we introduced the reconstruction network of UDIS to support other methods in image reconstruction. In addition, some methods (S+R, S+M, UDIS, UDIS-Variant, and CA-UDHN) cannot remove the regions outside the moving object based on the registration result. They require an additional segmentation method [2]. Therefore, we introduce MobileSAM [44], a fast segmentation method based on SAM [45] for these methods.

The evaluation metrics for reconstruction quality can be divided into two categories:

1. Subjective Quality Assessment: Following previous work [9], we use *error*, *failure*, and *success* to measure the subjective reconstruction quality. *Error* indicates the ratio of program crashes during computation (usually occurring in traditional methods); *failure* refers to the ratio of failed stitched images, including obvious deformation and intolerable artifacts; and *success* represents the ratio of successfully stitched images.
2. Objective Quality Assessment: Our dataset lacks labels, which prevents the use of full-reference evaluation metrics. Inspired by the No-Reference Image Quality Assessment (NR-IQA) [46], we propose incorporating PIQUE [47], NIQE [48], and DeQA [49] to measure the objective reconstruction quality. NR-IQA methods assess quality solely based on image features. Hence, they are highly flexible and widely applicable to various scenarios.

**Figure 6:**
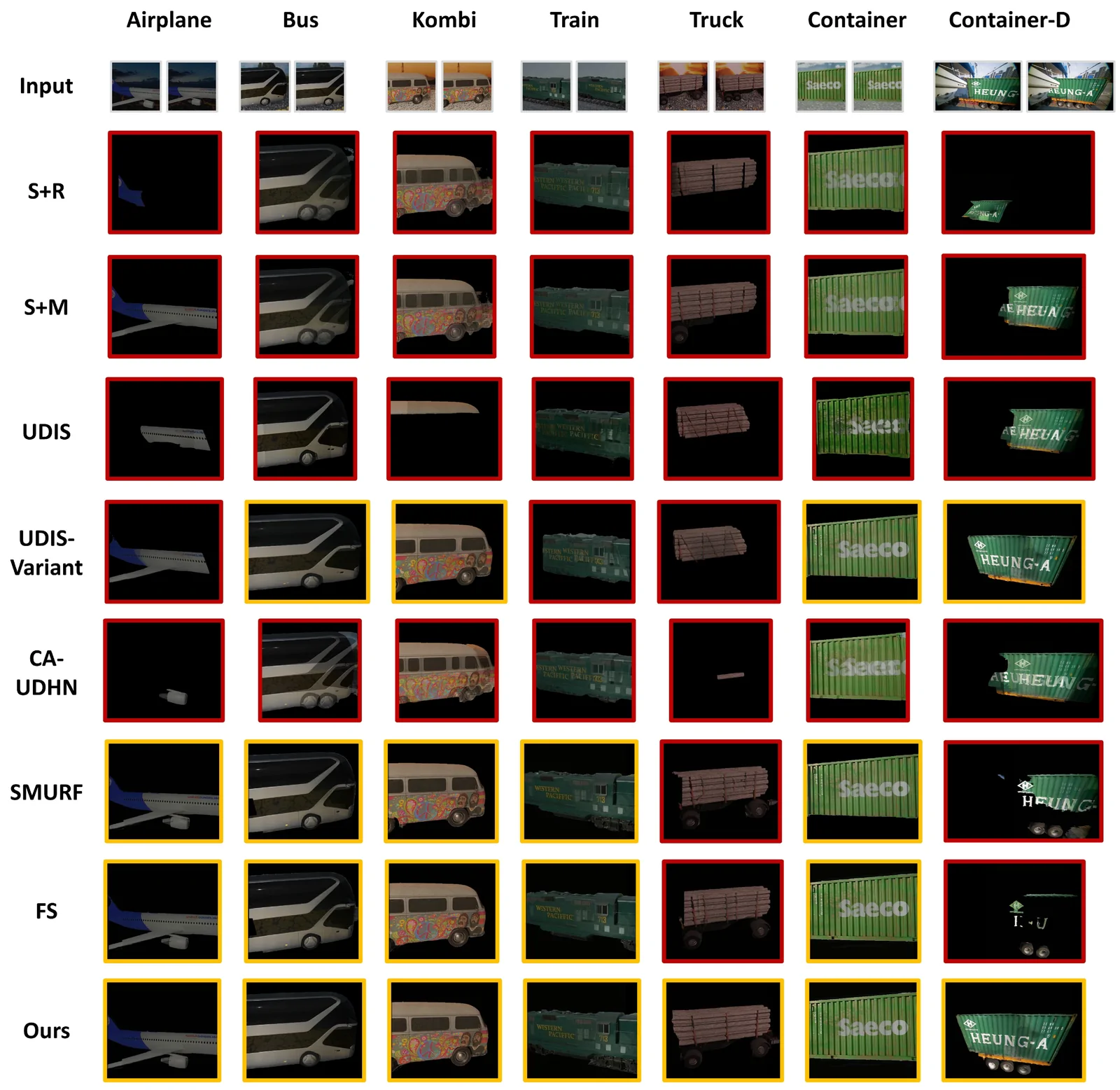
Visualization of reconstruction quality, where the red and yellow boxes indicate *failures* and *successes*, respectively.

To further intuitively verify the visual performance of different methods, we conduct a qualitative visual comparison of the stitched images, as illustrated in Figure 6. It can be clearly observed that the images stitched by the proposed method retain richer texture details and sharper edge contours. In contrast, the results obtained by other comparative methods suffer from obvious blurring artifacts, excessive smoothness and partial detail loss. Moreover, our reconstructed images present more natural visual effects with fewer noise residues and distortion phenomena, which demonstrates superior practical reconstruction capability and better visual perception quality.

Comparisons between our method and other methods are shown in Table 6 and Table 7. Notably, there is a substantial disparity between traditional and learning-based approaches, with the latter demonstrating greater robustness than the former.

### 4.5. Evaluation of Speed

We compare the speed of learning-based methods. Following previous work [44], the speed metric is defined as the inference time per image pair, as shown in Table 8. Specifically, in this part, we divide the stitching process into three stages: registration (Regis), segmentation (Seg), and reconstruction (Recon). Except for optical-flow-based methods (SMURF, FlowSupervisor, and our method), other methods require a segmentation model, so the segmentation time needs to be calculated. Additionally, SMURF and FlowSupervisor cannot estimate the bidirectional optical flow in a single inference; therefore, two inferences are required for registration.

### 4.6. Ablation Studies

In this section, we conduct ablation studies, which are divided into two parts.

#### 4.6.1. Ablation Study of Registration

For registration, we mainly evaluate the effect of semi-supervised learning. The proposed method is compared with a supervised technique, which uses only the pre-trained model, and an unsupervised technique, which fine-tunes the model using only the unsupervised loss. The results are shown in Table 9.

#### 4.6.2. Ablation Study of Reconstruction

For reconstruction, we mainly evaluate the effect of the multi-scale structure, as shown in Table 10. The multi-scale structure is used to improve image quality and does not directly affect the stitching success rate. Therefore, only PIQUE and NIQE are used in this ablation experiment. In addition, the reconstruction objective function is closely coupled with the network structure. Ablating the network structure is therefore equivalent to ablating the corresponding objective-function design, so the objective function is not ablated separately.

## 5. Conclusions

This paper proposes a semi-supervised deep image-stitching method for moving elongated objects. The proposed framework combines semi-supervised optical-flow registration, object-centric spatial transformation, and unsupervised multi-scale reconstruction. The registration stage estimates bidirectional optical flow and helps suppress foreground/background interference. The object-centric transformation module removes non-object regions and maps the input images onto a unified plane. The reconstruction stage then generates high-quality stitched images through multi-scale fusion and a reconstruction objective based on content, seam, perceptual, and consistency constraints. Experiments on the synthetic MEOIS-D dataset and the real-world Container-D dataset demonstrate the effectiveness of the proposed method. Compared with existing traditional and learning-based methods, the proposed method achieves strong overlap-region appearance consistency, improved reconstruction quality, and lower total inference time in the evaluated pipeline. Future work will focus on continuous multi-frame image stitching, tighter interaction between registration and reconstruction, and further acceleration for industrial deployment.

## Figures and Tables

**Table 1:** Displacement and rotation range settings for different overlap levels.

Overlap Level	Displacement	Rotation
**Large**	⌈length/15⌉∼⌈length/10⌉	−5°∼5°
**Middle**	⌈length/10⌉∼⌈length/8⌉	−10°∼10°
**Small**	⌈length/8⌉∼⌈length/5⌉	−15°∼15°

**Table 2:** Comparison of $P_{overlap}$ (↑) and $S_{overlap}$ (↑) according to overlap levels on MEOIS-D.

Overlap	Metric	Traditional		Learning-Based					
		S+R [11]	S+M [40]	UDIS [9]	UDIS-Variant [26]	CA-UDHN [41]	SMURF [42]	FS [33]	Ours
**Large**	$P_{overlap}$	21.16	22.10	25.45	26.22	20.31	26.58	26.67	**27.17**
	$S_{overlap}$	0.67	0.71	0.75	0.76	0.76	0.78	0.78	**0.81**
**Middle**	$P_{overlap}$	21.27	21.58	24.20	25.78	19.94	25.57	25.62	**26.28**
	$S_{overlap}$	0.66	0.69	0.71	0.77	0.75	0.76	0.77	**0.79**
**Small**	$P_{overlap}$	20.58	21.25	23.21	25.41	20.18	24.72	25.23	**25.62**
	$S_{overlap}$	0.66	0.67	0.68	0.75	0.74	0.76	0.76	**0.77**

**Table 3:** Comparison of $P_{overlap}$ (↑) and $S_{overlap}$ (↑) according to lighting conditions on MEOIS-D.

Lighting	Metric	Traditional		Learning-Based					
		S+R [11]	S+M [40]	UDIS [9]	UDIS-Variant [26]	CA-UDHN [41]	SMURF [42]	FS [33]	Ours
**Daytime**	$P_{overlap}$	20.74	21.24	22.36	23.52	19.96	23.63	23.72	**24.38**
	$S_{overlap}$	0.66	0.69	0.71	0.72	0.75	0.75	0.75	**0.78**
**Dusk**	$P_{overlap}$	20.92	21.78	24.26	25.41	20.01	25.65	25.57	**25.76**
	$S_{overlap}$	0.66	0.68	0.71	0.77	0.74	0.77	0.77	**0.78**
**Night**	$P_{overlap}$	21.35	21.89	26.25	28.46	20.46	27.58	28.22	**28.93**
	$S_{overlap}$	0.67	0.70	0.72	0.79	0.77	0.79	0.80	**0.82**

**Table 4:** Comparison of $P_{overlap}$ (↑) and $S_{overlap}$ (↑) according to object types on MEOIS-D, where Air and Con denote Airplane and Container.

Object	Metric	Traditional		Learning-Based					
		S+R [11]	S+M [40]	UDIS [9]	UDIS-Variant [26]	CA-UDHN [41]	SMURF [42]	FS [33]	Ours
**Air**	$P_{overlap}$	20.52	20.96	26.50	26.22	19.33	26.42	26.47	**26.67**
	$S_{overlap}$	0.66	0.67	0.82	0.82	0.74	0.82	0.82	**0.83**
**Bus**	$P_{overlap}$	21.64	22.74	24.13	**24.79**	20.24	24.35	24.42	24.52
	$S_{overlap}$	0.67	0.71	0.80	**0.83**	0.74	0.80	0.81	0.81
**Con**	$P_{overlap}$	20.85	21.16	23.62	27.39	20.63	26.52	27.59	**29.72**
	$S_{overlap}$	0.67	0.69	0.66	0.71	0.76	0.78	0.80	**0.85**
**Kombi**	$P_{overlap}$	20.98	21.39	23.00	25.89	19.80	**26.38**	26.19	26.13
	$S_{overlap}$	0.66	0.68	0.67	**0.83**	0.76	**0.83**	0.81	**0.83**
**Train**	$P_{overlap}$	20.72	22.03	25.56	25.60	20.24	25.78	25.84	**26.41**
	$S_{overlap}$	0.65	0.69	0.69	0.68	**0.75**	0.70	0.70	0.72
**Truck**	$P_{overlap}$	21.32	21.57	22.90	**24.91**	20.62	24.27	24.53	24.68
	$S_{overlap}$	0.65	0.68	0.65	0.70	**0.74**	0.68	0.68	0.71

**Table 5:** Comparison of average $P_{overlap}$ (↑) and $S_{overlap}$ (↑), where M-D and C-D denote the MEOIS-D and Container-D datasets.

Dataset	Metric	Traditional		Learning-Based					
		S+R [11]	S+M [40]	UDIS [9]	UDIS-Variant [26]	CA-UDHN [41]	SMURF [42]	FS [33]	Ours
**M-D**	$P_{overlap}$	21.00	21.64	24.29	25.80	20.14	25.62	25.84	**26.36**
	$S_{overlap}$	0.66	0.69	0.72	0.76	0.75	0.77	0.77	**0.79**
**C-D**	$P_{overlap}$	18.35	18.84	21.13	22.09	16.98	22.17	22.34	**23.82**
	$S_{overlap}$	0.62	0.63	0.68	0.68	0.69	0.72	0.73	**0.75**

**Table 6:** Comparison results on MEOIS-D.

Metric	Traditional		Learning-Based					
	S+R [11]	S+M [40]	UDIS [9]	UDIS-Variant [26]	CA-UDHN [41]	SMURF [42]	FS [33]	Ours
*Error* ↓	0.15	0.08	**0**	**0**	**0**	**0**	**0**	**0**
*Failure*↓	0.68	0.54	0.57	0.26	0.61	0.16	0.15	**0.08**
*Success*↑	0.17	0.38	0.43	0.74	0.39	0.84	0.85	**0.92**
PIQUE ↓	37.71	37.48	39.19	40.10	38.48	30.65	31.61	**28.41**
NIQE ↓	21.81	21.83	22.44	21.63	21.94	11.58	11.67	**10.37**
DeQA ↑	2.49	2.94	2.94	3.28	3.13	3.26	2.98	**3.52**

**Table 7:** Comparison results on Container-D.

Metric	Traditional		Learning-Based					
	S+R [11]	S+M [40]	UDIS [9]	UDIS-Variant [26]	CA-UDHN [41]	SMURF [42]	FS [33]	Ours
*Error* ↓	0.24	0.13	**0**	**0**	**0**	**0**	**0**	**0**
*Failure*↓	0.63	0.54	0.62	0.38	0.68	0.24	0.21	**0.13**
*Success*↑	0.13	0.33	0.38	0.62	0.32	0.76	0.79	**0.87**
PIQUE ↓	35.69	35.74	27.56	27.75	27.04	**13.09**	13.97	13.20
NIQE ↓	21.92	21.85	21.94	22.59	22.61	12.59	13.02	**12.05**
DeQA ↑	2.49	2.94	2.98	3.32	3.38	3.46	3.44	**3.74**

**Table 8:** Comparative experiments of speed (second ↓). The best performers are marked in bold.

Method	Regis	Seg	Recon	Total
**UDIS [9]**	0.09	4	0.18	4.27
**UDIS-Variant [26]**	0.06	4	0.12	4.18
**CA-UDHN [41]**	0.05	4	0.18	4.23
**SMURF [42]**	1.6	-	0.18	1.78
**FS [33]**	1.6	-	0.18	1.78
**Ours**	1.5	-	0.17	**1.67**

**Table 9:** Ablation study of registration. The best performers are marked in bold.

Supervised	Unsupervised	Semi-Supervised	$P_{\mathrm{overlap}}↑$	$S_{\mathrm{overlap}}↑$
*√*			25.17	0.68
*√*	*√*		24.39	0.71
*√*		*√*	**26.36**	**0.79**

**Table 10:** Ablation study of reconstruction. The best performers are marked in bold.

Single-Scale	Two-Scale	Three-Scale	PIQUE ↓	NIQE ↓	DeQA ↑
*√*			30.52	11.49	3.28
*√*	*√*		29.85	11.05	3.44
*√*	*√*	*√*	**28.41**	**10.37**	**3.52**

## Data Availability

The MEOIS-D dataset is available from the corresponding author upon reasonable request. The Container-D dataset contains internal enterprise data and is not publicly available.
